# Modern three-dimensional digital methods for studying locomotor biomechanics in tetrapods

**DOI:** 10.1242/jeb.245132

**Published:** 2023-02-22

**Authors:** Oliver E. Demuth, Eva Herbst, Delyle T. Polet, Ashleigh L. A. Wiseman, John R. Hutchinson

**Affiliations:** ^1^ Department of Earth Sciences, University of Cambridge, Cambridge, CB2 3EQ, UK; ^2^ Palaeontological Institute and Museum, University of Zurich, 8006 Zürich, Switzerland; ^3^ Structure and Motion Laboratory, Department of Comparative Biomedical Sciences, Royal Veterinary College, North Mymms, AL9 7TA, UK; ^4^ McDonald Institute for Archaeological Research, University of Cambridge, Cambridge, CB2 3ER, UK

**Keywords:** Animation, Bone, Dynamics, Gait, Muscle, Optimization

## Abstract

Here, we review the modern interface of three-dimensional (3D) empirical (e.g. motion capture) and theoretical (e.g. modelling and simulation) approaches to the study of terrestrial locomotion using appendages in tetrapod vertebrates. These tools span a spectrum from more empirical approaches such as XROMM, to potentially more intermediate approaches such as finite element analysis, to more theoretical approaches such as dynamic musculoskeletal simulations or conceptual models. These methods have much in common beyond the importance of 3D digital technologies, and are powerfully synergistic when integrated, opening a wide range of hypotheses that can be tested. We discuss the pitfalls and challenges of these 3D methods, leading to consideration of the problems and potential in their current and future usage. The tools (hardware and software) and approaches (e.g. methods for using hardware and software) in the 3D analysis of tetrapod locomotion have matured to the point where now we can use this integration to answer questions we could never have tackled 20 years ago, and apply insights gleaned from them to other fields.

## Teaser

**Summary:** Three-dimensional digital methods useful for the study of tetrapod locomotor biomechanics have matured in recent decades. Here, we summarise their progress, relevance and potential.

## Introduction

Scientists studying organismal biomechanics at the musculoskeletal level (and neural control thereof) today are working in a new age of modern methods at the interface of empirical and theoretical approaches to movement, which were unavailable or very immature even 20 years ago. Examples of the former include XROMM (X-ray reconstruction of moving morphology; see Glossary; Brainerd et al., 2010) and the latter includes predictive simulation (see Glossary; e.g. Falisse et al., 2019). These techniques have much in common, particularly the importance of three-dimensional (3D) digital technologies and the ability to test hypotheses about comparative locomotor biomechanics in unprecedented spatial and temporal resolution. By ‘digital’ here, we refer to the central importance of software for processing those complex 3D data. Computational power and software sophistication are central in these digital technologies because they enable analyses that could not realistically be done manually in a real-world setting. Furthermore, these 3D digital methods are visual in nature, producing valuable data on, and compelling visualisations of, 3D locomotor dynamics. Because of this digital nature that these approaches share, they can be powerful together, giving major insights into the kinematics, kinetics and control of locomotion.

One might ask, why bother using 3D digital methods at all? The simple answer is that they can more objectively handle complex computational problems whilst visualising organismal morphology and motion in realistic detail. That detail allows answering biological questions about how organisms locomote (using mechanisms that may be complex) in the real, 3D world; and how real, 3D (often complex) morphology plays a role in locomotor behaviours. However, 3D approaches (and more complex varieties of 3D methods) need not be used simply because they exist. The benefits should be weighed considering their ability to answer research questions, and their cost in terms of time, accuracy and other challenges (e.g. Hicks et al., 2015). As an example, if the research question is about how a muscle functions in one plane, a more 2D approach might provide sufficient fidelity, although this would introduce assumptions that dynamics in other planes are of negligible importance. Yet a 3D approach might not provide such outstanding benefits that they justify the effort, or might be impractical in terms of experimental data collection. The answer to this conundrum may, admittedly, lie in researcher preferences and traditions– not just purely practical issues – and thus there may be no one ‘correct’ answer for a given research question.

Here, we focus on how the biomechanical application of 3D digital approaches enables more powerful tests of how tetrapod vertebrates use their appendages to locomote (both support and motion) on land. We do not cover feeding, breathing, vertebral motions, flight and swimming, etc., although these behaviours have ideas in common with (and sources of inspiration for) our topic. Despite its high relevance, space constraints prevent us from truly covering the huge amount of research on human locomotion. We also do not review biorobotics in depth. There is tremendous relevance of research on arthropod locomotion, but again we cannot adequately review that field here. Nor do we cover limbless locomotion or the role of the axial column in tetrapod movement. We briefly address sensitivity analysis (see Glossary), and especially ‘validation’ (Hutchinson, 2012; Hicks et al., 2015) or model evaluation. We also discuss how comparative, evolutionary and palaeobiological studies have benefitted, and reciprocally moved the state of the art forwards, for the methods we focus on.

We begin by considering 3D digital tools such as XROMM and musculoskeletal modelling; such tools may be used in empirical contexts to address questions about locomotor or appendicular dynamics. We then explore the spectrum (explained below) of ways that researchers in biomechanics can use digital methods to understand locomotor mechanisms, such as finite element analysis (FEA; see Glossary) and dynamic simulation. This spectrum proceeds from more empirical toward more theoretical analyses, but all analyses here considered arguably involve a form of ‘model’ (see Glossary) that their digital methods visually represent. Next, we investigate how integration of digital tools across this spectrum achieves novel, exciting understanding of motion. Finally, we explore the pitfalls and challenges involved in these approaches; and current frontiers at the cutting edge of using 3D digital tools and methods in tetrapod locomotor biomechanics. Our review demonstrates how tools have matured to the point where now we can use them in isolation or integration to answer fundamental questions we never could have tackled two decades ago.

**Glossary**

**Conceptual model**

An abstraction of an organism to a small number of parameters to investigate fundamental functional principles.

**Data overfitting**

When a model is adjusted to match its training or validation set too closely, and is thus unable to generalise to new datasets.

**Degree(s) of freedom (DOF)**

The number of parameters that can vary in a system (e.g. axes of joint motion).

**Finite element analysis (FEA)**

Estimating stress or strain using smaller components subjected to load(s).

**Forward dynamic simulation**

Simulation that solves a differential equation of a system's physics over incremental timesteps.

**Forward kinematics**

Using joint angles to estimate end (e.g. foot) positions.

**Inverse dynamic/static simulation**

Simulation that solves joint moments (and potentially muscle forces and activations) from input kinematics and kinetics. In the static case, static equilibrium is assumed.

**Inverse kinematics**

Using an endpoint of a series of segments (e.g. foot) to estimate joint angles.

**Multi-body dynamics analysis (MDA)**

Rigid body mechanics.

**Model**

A representation of reality, used to understand reality.

**Model evaluation**

‘Validation’, i.e. testing how well theoretical predictions match empirical data.

**Moment arm**

Leverage of a force around a rotational centre.

**Musculoskeletal model**

A skeletal framework around which the geometry of muscle–tendon units is positioned.

**Optimal control**

A set of methods to find inputs to a time-dependent system that minimises an objective function.

**Precision**

The reproducibility, or repeated variation, of a given measurement.

**Predictive simulation**

Estimating system outputs using only inputs of optimisation criteria and constraints.

**Robustness**

How changes in model inputs influence output fidelity to empirical data.

**Sensitivity analysis**

Varying model/simulation input parameters or assumptions to quantify variation of the output data.

**Synergistic approach**

Combination of empirical and dynamic simulation data, enhanced by the benefits of both.

**Tracking simulation**

Conducting a simulation with an objective to best match input empirical data.

**Verification**

Testing the mathematical validity of the design of a model or simulation.

**XROMM**

X-ray reconstruction of moving morphology: animation of a 3D skeletal marionette using biplanar X-ray video data.

## Digital tools for empirical data

Dynamic assessment of 3D skeletal motion is key to the understanding of how animals move and how their movement might be affected as a result of injury or disease. Such kinematic data can be obtained with high precision using biplanar fluoroscopy (*in vivo* and/or *ex vivo* X-ray videos) combined with 3D bone geometry (XROMM); or motion capture. A researcher must first capture kinematic data and collect high quality 3D data [e.g. from computed tomography (CT) or magnetic resonance imaging (MRI) scans] of that specimen either before or after experimentation. These scans provide 3D bone geometries and the locations of any implanted markers to be tracked. Fluoromicrometry offers an analogue to XROMM for studying soft tissue kinematics, and empirical data from anatomy and kinematics may be used for building and analysing musculoskeletal models. Together, realistic 3D kinematic data produced by these tools also can be inputs that improve the accuracy of more theoretical models and simulations, or that can be used to evaluate how accuracy and any uncertainties impact the ability to answer a research question (see further below).

### XROMM

**Fig. 1.**
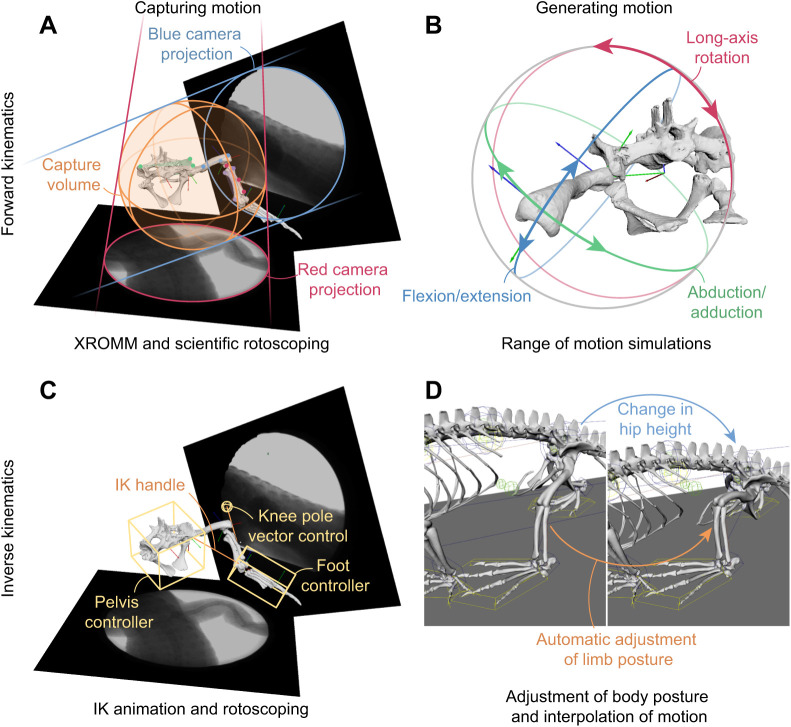
**The capture and generation of motion.** Skeletal motion can be captured through matching the bones to X-ray shadows using XROMM (A) or inverse kinematic (IK) animation tools (C). Motion can be quantified either through forward kinematic simulations (B) or alternatively, through adjustment of individual parameters, such as hip height, new hypothetical motion can be generated in inverse kinematic setups (D). Additionally, inverse kinematic tools can be used to interpolate motion that could not be directly captured, e.g. if the specimen leaves the capture volume. Nile crocodile bone models (A–C) and X-ray data (A,C) from Wiseman et al. (2021); *Caiman* model (D) from Nyakatura and Demuth (2019).

In one version of XROMM (Fig. 1), small beads are implanted into bones (Tashman and Anderst, 2003), which are then automatically tracked, and the bones' 3D positions are linked to the beads' positions by skeletal morphological data (Brainerd et al., 2010). Alternatively, XROMM data can be manually or semi-automatically rotoscoped by matching bones to their respective X-ray shadows using a hierarchical joint marionette (‘scientific rotoscoping’; Gatesy et al., 2010) when no or too few implanted markers are present. In this case, the proximal or distal segments/joint positions and orientations drive the positions and orientations of the connected upstream or downstream segments in the hierarchy (forward kinematics or inverse kinematics; see Glossary), and respective segments can be further refined based on this ‘initial guess’ (e.g. Fischer et al., 2010; Nyakatura and Demuth, 2019; Turner et al., 2020; Turner and Gatesy, 2021; Zwafing et al., 2021; Herbst et al., 2022a; Wiseman et al., 2022a). There is a rich literature on the usage of 3D digital kinematic data such as XROMM for studying terrestrial legged locomotion in tetrapods *in vivo* (e.g. Nyakatura and Fischer, 2010; Baier and Gatesy, 2013; Nyakatura et al., 2014; Bonnan et al., 2016; Heers et al., 2016; Mayerl et al., 2016; Kambic et al., 2017; Lin et al., 2019; Turner and Gatesy, 2021), which we cannot adequately cover here. XROMM data allow the calculation of joint kinematics using anatomically relevant coordinate systems which define the joint centres (e.g. Kambic et al., 2014; Gatesy et al., 2022). We consider XROMM data as a form of ‘model’ (but more on the empirical end of the spectrum, away from pure theory), because they are an abstraction of the empirical (X-ray video and 3D scan) data. The assumptions and simplifications of XROMM include matching jointed marionettes to biplanar 2D images, 3D bone segmentation and meshing, the fundamental assumption that bones are perfectly rigid objects, and the frequent simplification of joint degrees of freedom (DOF; see Glossary); or the number of joints connecting segments.

### Motion capture

Motion capture uses special cameras to digitally capture the 3D positions of markers on a subject. Software then reconstructs the 3D motions of the markers and the segments they are placed on. Motion capture may also be markerless (e.g. Mathis et al., 2018; Moore et al., 2022). Whilst the positional marker data are truly empirical, the resulting kinematic data for segments are a form of ‘model’, for reasons similar to those for XROMM. However, motion capture also involves the assumption that skin-attached markers accurately reflect underlying skeletal 3D positions – at best only somewhat true – and this is one reason why XROMM typically is considered more accurate (Cerveri et al., 2005; Shultz et al., 2011; Moore et al., 2022). However, motion capture has the benefits that it can be readily available (and more affordable than XROMM), is easily used in most laboratory settings, tends to provide much larger capture volumes, and involves no radiation dosage, along with being less invasive. For *ex vivo* experiments where soft tissue is removed (e.g. Arnold et al., 2014), motion capture avoids the issues of movement between skin/markers and skeleton. Markers can be attached to the bones or, for small specimens, can be attached elsewhere and the 3D data transformed to a joint-centric motion (e.g. Stowers et al., 2017; Manafzadeh, 2020; Herbst et al., 2022a). There have been a tremendous number of 3D motion capture studies in the field, which we cannot do justice to here.

### *Ex vivo* and virtual experiments

Whilst XROMM facilitates a direct observation of the skeletal kinematics of an animal, it is inherently limited to the capture volume and the behaviour an animal exhibits within it (Fig. 1A). Motion experiments involving cadavers allow measurement of the passive 3D mobility repertoire (Arnold et al., 2014; Stowers et al., 2017; Manafzadeh, 2020; Regnault et al., 2021). Digital animation tools permit the manipulation of a skeleton via its joint marionette, where the user has full control over the position and orientation of individual body segments. This allows one to produce any behaviour imaginable, ranging from behavioural reconstructions that are informed by motion data (Lee et al., 2020) to excluding extreme and unlikely (Nyakatura et al., 2019; Zwafing et al., 2021), or even implausible and impossible bone orientations (Manafzadeh and Padian, 2018; Fig. 1B). Such virtual experiments can, therefore, examine functional limits constrained solely by skeletal morphology, and generate new and novel skeletal movements that cannot be otherwise observed. These experiments can be performed using inverse kinematics animation tools such as inverse kinematics solvers (e.g. Nyakatura et al., 2019; Wiseman et al., 2021; Fig. 1C) whereby the proximal and distal limb elements are manually positioned and the orientations of remaining elements are interpolated (Wiseman et al., 2022a; Fig. 1D). Alternatively, forward kinematics animations can be created to systematically sample joint poses to estimate the overall mobility of a joint (e.g. Manafzadeh and Padian, 2018), which could otherwise not be practically observed *in vivo*. Additional constraints can be incorporated into such animations to force joint articulation (Lee et al., 2020; Jones et al., 2021; Bishop et al., 2022).

### Fluoromicrometry

Like XROMM, biplanar fluoroscopy with 3D anatomical data can reveal the roles of soft tissues during locomotion (i.e. ‘fluoromicrometry’; Camp et al., 2016; see also Regnault et al., 2021). Tsai et al. (2019) applied XROMM to walking alligators to obtain skeletal kinematics, but then combined these 3D data with 3D morphology of articular cartilage, ligaments and other tissues around the hip joint to estimate deformations of those tissues and their roles in joint motion. Astley and Roberts (2012) used XROMM to obtain skeletal kinematics and tendon strain during hopping in frogs, revealing how the plantaris muscle–tendon unit (MTU) acted as a catapult mechanism around the ankle joint. Similarly, Arellano et al. (2019) integrated XROMM skeletal kinematics with changes of M. gastrocnemius lateralis fibre length and aponeurosis length and width in wild turkeys during landing and jumping, showing that aponeurosis width had negative effects on aponeurosis stiffness in the longitudinal direction, and thereby how springlike the MTU was. Konow et al. (2020) used fluoromicrometry of the M. gastrocnemius medialis in rats to show that, as slope and gait change during locomotion, muscle fibre and tendon lengths and muscle width increase, whilst muscle thickness decreases. XROMM with soft tissue implantations has also been used in veterinary contexts, such as the superficial digital flexor tendons in the forelimbs of ponies, revealing up to ∼6% strain *in vivo* during trotting (Wagner et al., 2021). Whereas Allen et al. (2017) principally measured skeletal kinematics *in vivo* via XROMM in the guineafowl hindlimb, they used these data to infer the mechanical leverage of the patellar sesamoid and thereby its influence on knee extensor muscle actions. Regnault et al. (2017) conducted similar inquiries with the ostrich knee joint and its ‘double patella’ system *ex vivo*.

### Musculoskeletal modelling: a more empirical basis

**Fig. 2.**
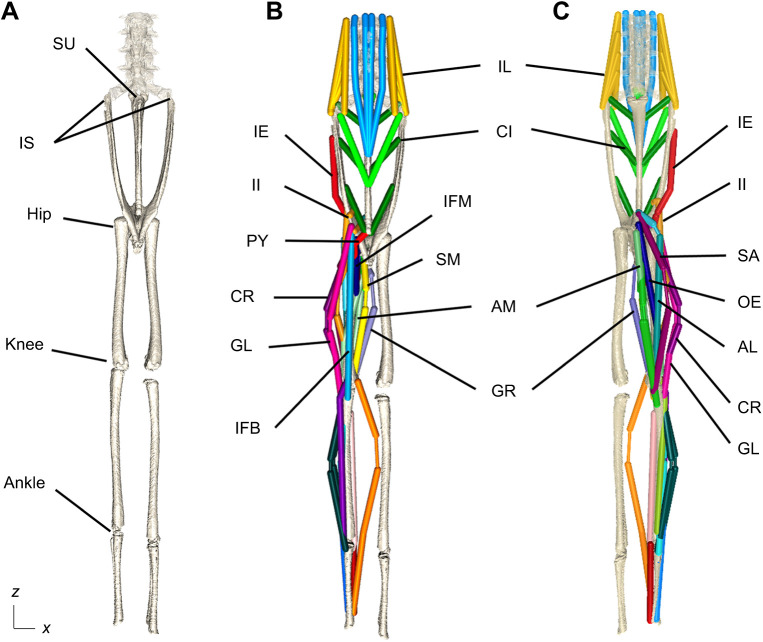
**Sample musculoskeletal model of the hindlimbs of a red-legged frog, *Kassina*.** The model is shown in dorsal (A,B) and ventral (C) views (from Collings et al., 2022). Limbs are in a straightened reference pose. (A) Joints: SU, sacrourostylic; IS, iliosacral, with axes shown (*x*, flexion/extension; *z*, long-axis rotation). (B,C) Muscles: AL, M. adductor longus; AM, M. adductor magnus; CI, M. coccygeoiliacus; CR, M. cruralis; GL, M. gluteus maximus; GR, M. gracilis major; IE, M. iliacus externus; IFB, M. iliofibularis; IFM, M. ilofemoralis; II, M. iliacus internus; IL, M. iliolumbaris; OE, M. obturator externus; PY, M. pyriformis; SA, M. sartorius; SM, M. semimembranosus. Creative Commons Attribution CC-BY license.

3D digital tools can give new understanding of musculotendinous (henceforth ‘muscle’) function during locomotion in extant tetrapods, typically beginning with a 3D skeletal marionette as in XROMM, but then adding soft tissues (Fig. 2). These musculoskeletal models (see Glossary) normally are grounded in empirical data such as dissections or 3D imaging of soft tissue morphology (e.g. Brown et al., 2003; Nagano et al., 2005; Zarucco et al., 2006; Hutchinson et al., 2015; Charles et al., 2016; Regnault and Pierce, 2018; Sullivan et al., 2019; Bishop et al., 2021b; Wiseman et al., 2021; Collings et al., 2022), or a 3D polygonal modelling approach (Demuth et al., 2022a; Herbst et al., 2022b), and are sometimes placed within a phylogenetic context (Brocklehurst et al., 2022; Löffler et al., 2022). Musculature must be reconstructed if the research question pertains to the moment arms (see Glossary), moment-generating capacity and/or activation of specific muscles/muscle groups. Muscle architectural data (e.g. fibre length, pennation angle, tendon slack length) can be obtained from dissection data, or simplified into whole MTUs (e.g. Bishop et al., 2021a). Such muscle data enable the creation of subject-specific theoretical musculoskeletal models (see below).

Muscle modelling tools, often combined with data from or estimates of joint mobility, can be used to test how muscle moment arms depend on limb orientation (joint angles/kinematics). Empirical data from experimental measurements of muscle moment arms via ‘tendon travel’ (An et al., 1984; Lieber, 1997; Cox et al., 2019), fluoromicrometry, or other methods, including more theoretical ones (e.g. Alexander and Dimery, 1985; Sherman et al., 2013; Allen et al., 2017; Regnault et al., 2017; Young et al., 2019), are highly valuable in conjunction with these studies, to evaluate their accuracy. These studies of muscle leverage versus limb orientation often address a fundamental principle of great relevance to locomotor biomechanics: how closely matched are ‘optimal’ moment arms to limb orientation such as during maximal ground reaction forces (e.g. Fujiwara, 2018)? Empirically based 3D studies on this topic tend to have fairly mixed or nuanced results and conclusions (Kargo and Rome, 2002; Hutchinson et al., 2015; Cox et al., 2019; Wiseman et al., 2021). Hence, assumptions that maximal muscle moment arms indicate ‘optimal posture’ remain on somewhat shaky ground. However, quantification of how moment arms and limb orientation covary is still valuable, giving insight into muscle function.

## Spectrum of digital modelling methods: toward theoretical approaches

### Empirical-to-theoretical spectrum

**Fig. 3.**
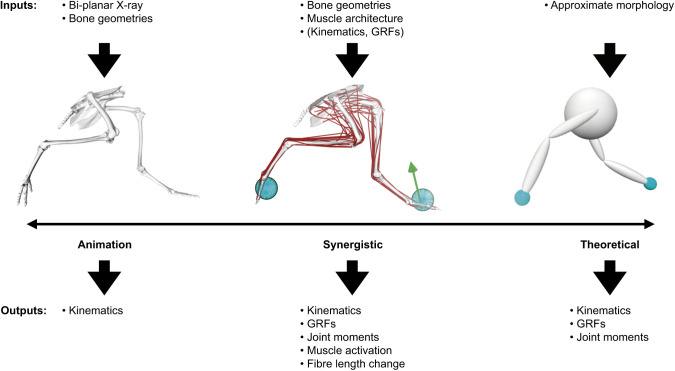
**Digital tools lie on a spectrum, from more empirical animation methods to theoretical and conceptual models.** Left: animation methods, such as XROMM, put measured data into a 3D visualisation, where component interaction can be observed. Shown here is a kinematic tinamou model, described by Bishop et al. (2021b), where the positions of limb bones were derived from XROMM. For animation methods, the required inputs tend to be large compared to the outputs. Right: theoretical methods can involve high levels of abstraction, generating many outputs with few empirical measurements. For example, the tinamou may be modelled as a simple sphere with two-joint legs (here, ‘hips’ and ‘knees’). Such a model can be integrated with predictive simulation to study control, stability, economy or other aspects of locomotion from a broad (if not precise) perspective. Middle: synergistic methods combine empirical data with theoretical models to produce rich datasets. Bishop et al. (2021b) combined XROMM data with measured ground reaction forces (GRFs), and modelled the line of action of various muscles. Using inverse tracking simulations (see Glossary), they could predict muscle fibre length changes and activations, which could not be measured experimentally. While synergistic methods require more empirical inputs, they have the potential to generate much more (unobservable) data by integrating these inputs though model-based analyses.

Fig. 3 shows how 3D modelling approaches fall on a spectrum from empirical methods (discussed above) to purely theoretical models. In a more empirical example, XROMM was used to reconstruct a tinamou during running (Bishop et al., 2021b). Biplanar X-ray and bone geometries formed the input, with 3D kinematics being an output. A ‘synergistic’ approach (see Glossary) used by Bishop et al. (2021b) combined XROMM data with abstracted muscle architecture and optimisation techniques to explore joint moments, muscle activations and fibre length changes during locomotion. Synergistic models combine empirical and theoretical approaches to generate rich datasets that allow researchers to infer unmeasurable data, thereby more holistically testing hypotheses on locomotor biomechanics or control. More theoretical models enable researchers to (1) infer data that are difficult to collect empirically, which (2) tests the effects of morphology on locomotor mechanisms. Methods for such models and simulations include musculoskeletal modelling (Fig. 2; e.g. of muscle moment arms as per extant taxa above; but based on minimal empirical data), finite element analysis, inverse and forward dynamic simulation, soft body dynamics, dynamic simulation of particles, and combinations of the above. In some cases, more theoretical, even conceptual models (see Glossary) can be far-removed from the organism; the tinamou might be abstracted as a simple volume connected to two-joint legs (Fig. 3). Such a model can be explored with theoretical techniques to test control strategies that can be compared (usually in qualitative terms) with organismal behaviour.

### Conceptual models

Conceptual – or theoretical (Fig. 3) – models, which greatly abstract the organism to salient features of interest, are commonplace in studies of organismal control of movement (e.g. Alexander, 1989; Blickhan, 1989; Farley et al., 1993; Seipel et al., 2017). Most examples are 2D; because of the mostly parasagittal nature of avian and mammalian legged locomotion, the vast majority of conceptual models abstract the organism to motion in the sagittal plane (with a smaller number focusing solely on motion in other planes). The parasagittal approximation is an obvious and natural level of abstraction, but it has the potential to hide important dynamic effects (e.g. Kambic et al., 2014, 2017), and cannot address several important open questions in locomotor biomechanics – such as the distribution of diagonal sequence gaits (Cartmill et al., 2002), or the functional significance of pacing (Janis et al., 2002).

While comparatively rare, other conceptual studies explore the 3D nature of locomotion. Usherwood and Davies (2017) explored work-minimising slow quadrupedal walking with a point mass 3D model. The model had only a few input parameters: duty factor (tied to speed), phase and three parameters affecting ground reaction force shape. Yet, a common pattern of increasing phase with increased speed emerged from the model, implying the perceived change in control reduced the cost of locomotion. Usherwood and Smith (2018) applied a 3D geometric model to explain the phase sequences of slow grazing gaits, with a tentative explanation for diagonal sequence gaits in primates. They later developed a robot based on the concept (Smith and Usherwood, 2020).

Passive dynamic conceptual models ignore muscular actuation and instead focus on the dynamics of locomotion under the influence of gravity. Remy et al. (2010) produced a passive dynamic quadrupedal 3D model with 1 DOF rigid legs. Whilst they found no difference in economy or stability between lateral and diagonal sequence gaits, they found that increased leg spacing stabilised the gaits and affected limb phase, at the expense of economy. Other studies have focused on recovery from disturbance at a conceptual level, where the spring-loaded inverted pendulum (SLIP) model is a common conceptual basis. Whilst many 2D studies have examined perturbation rejection in animals using SLIP models (e.g. Blum et al., 2014), and 3D SLIP-based model control has been applied to human running (e.g. Peuker et al., 2012) and to quadrupedal robots (e.g. Han et al., 2022), only one study to our knowledge has examined the stability of a 3D SLIP model in a non-human tetrapod (a kangaroo-inspired hopper; Seipel and Holmes, 2006). The usage of 3D SLIP models to test hypotheses about 3D locomotor control and stability is an area ripe for exploration.

### Musculoskeletal modelling: a more theoretical basis

Theoretical models can also be used to probe questions that are experimentally difficult or impossible to undertake. Because more empirical approaches are impossible for extinct tetrapods, these models have been very popular for estimating locomotor function in palaeobiology, and thereby there has been much progress in using the methods themselves. Modelling 3D muscle moment arms (and possibly maximal moment-generating capacity) has become popular for understanding locomotor function in extinct taxa, especially dinosaurs and other archosaurs (e.g. Hutchinson et al., 2005; Bates et al., 2015; Brassey et al., 2017; Otero et al., 2017; Cuff et al., 2022) but also in other extinct tetrapods (e.g. Molnar et al., 2021). Here, the core input empirical data tends to be 3D skeletal morphology via scanning, which, if not well preserved, can be scaled and composited into one individual (e.g. Demuth et al., 2020; Molnar et al., 2021), or represented as an idealised model of a species (Demuth et al., 2022b). Soft tissue anatomy is inferred somewhat directly from ‘muscle scars’ on bones that are osteological correlates of soft tissue attachments or (more indirectly) relative positions in extant relatives; this inferential approach uses the ‘extant phylogenetic bracket’ (Witmer, 1995; also see Bishop et al., 2021a) or analogues (Demuth et al., 2022a). These methods can answer how 3D muscle function evolved across vast macroevolutionary scales and key morphological transitions (e.g. Bates et al., 2015; Allen et al., 2021; Molnar et al., 2021; Brocklehurst et al., 2022; Cuff et al., 2022).

Joint mobility constrains the range of axial and/or appendicular kinematics. Quantifying these kinematic limits is important for deciphering evolutionary patterns in major transformations of axial and/or limb function. Digital tools enable the quantification of 3D joint mobility (Fig. 1B) not only in extant taxa but also in extinct ones (Pierce et al., 2012; Nyakatura et al., 2015; Manafzadeh and Padian, 2018; Demuth et al., 2020; Richards et al., 2021; Herbst et al., 2022c; Wiseman et al., 2022b) where, unlike in extant animals, the joint movement cannot directly be observed or otherwise estimated. The osteological range of motion (ROM) is influenced by the thickness of articular cartilage (permitting more or less motion depending on joint spacing), and by the accuracy of the joint centre position estimation (Demuth et al., 2020; Herbst et al., 2022c; Wiseman et al., 2022b). Because of the complex interplay of different DOF during motion (Kambic et al., 2014; 2017), single-axis estimates have been deemed unreliable and multi-axis mobility needs to be assessed (Manafzadeh and Padian, 2018). However, considering all rotational DOF together might even be insufficient as a result of translational movement present in the joints of extant taxa (Manafzadeh and Gatesy, 2021, 2022) or uncertainties in the joint centre position of extinct taxa (Wiseman et al., 2022b). Although the inclusion of translations allows studies to capture all 3D mobility present in a joint, these simulations can overestimate the true mobility that would otherwise be restricted by soft tissues (Arnold et al., 2014). Recent advances in simulating ligaments and other soft tissue constraints (Manafzadeh and Padian, 2018; Lee et al., 2020; Jones et al., 2021; Griffin et al., 2022; Bishop et al., 2022) can narrow down the osteological joint mobility to more accurately estimate true mobility. Joint mobility estimates can be combined with musculoskeletal models to compute muscle moment arms across the whole set of viable limb postures and draw evolutionary inferences (Brocklehurst et al., 2022).

### Finite element analysis

**Fig. 4.**
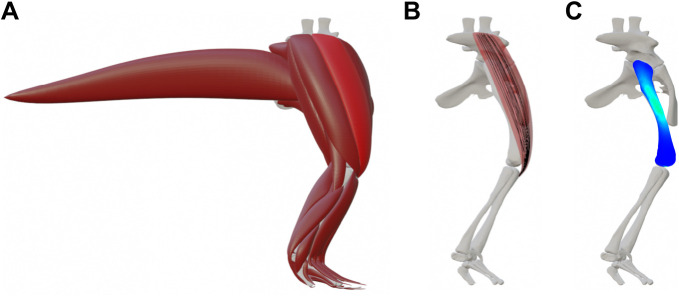
**Different methods for muscle modelling and usage in finite element modelling.** (A) 3D muscles were reconstructed for the right hindlimb of Triassic reptile *Euparkeria* (bone images from Demuth et al., 2020)*.* (B) Muscles can be decomposed into curved strands (black lines) or even as a 3D finite element mesh with these lines as embedded fibres to model lines of actions and deformations during movement. (C) Finite element analysis (FEA) can estimate the stresses or strains in bones (heat map indicates stresses; the low-to-high stress colour spectrum is cool/blue to hot/red). Muscle strands were created using the plugin of J. Hereš (https://doi.org/10.5281/zenodo.7100418), based on Kohout and Kukačka (2014) and Modenese and Kohout (2020).

Finite element analysis allows investigation of stress and strain distributions when forces are applied to an object. FEA can act as a bridge between studying various aspects of bone structure (microstructure, material properties and overall bone shape) and kinetics (Fig. 4). It can be used to examine the effects of bone and joint morphologies and loading environment on stress distributions, for example, to investigate functional implications of certain bone shapes. FEA involves dividing the object into polygonal elements and assigning material properties to the object to model its physical behaviour (Richmond et al., 2005; Rayfield, 2007; Bright, 2014; Blasi-Toccacceli et al., 2022). Keeffe and Blackburn (2022) used FEA to investigate the mechanical implications of radioulnar fusion in frogs, concluding that the semi-fused condition present in frogs is associated with smaller von Mises stress and higher bending resistance than in unfused or completely fused conditions. FEA of the carpal bones of a chimpanzee revealed that scaphoid–centrale fusion has lower stresses in knuckle walking than lack of fusion, supporting this fusion's role in that behaviour and its evolution (Püschel et al., 2020). FEA of trabecular bone across a range of loadings in the proximal femur of two primate species showed similar stress and strain distributions, revealing how bone strength can be preserved despite differences in 3D trabecular morphology (Ryan and van Rietbergen, 2005).

FEA can also be used to address developmental questions including the evolution of joint or bone shapes, because bony morphology reflects loading regime. Carter et al. (1987) proposed that hydrostatic compression decelerates and intermittent high shear stress accelerates endochondral growth and ossification. Using an FEA model subjected to such mechanistic stresses as well as growth and remodelling, Stevens et al. (1999) were able to create a model of endochondral growth that reflected key prenatal and postnatal developmental mechanisms. Chadwick et al. (2014, 2015) combined 3D *ex vivo* morphology obtained via CT and MRI scans with FEA to test how mechanobiological loading environments might relate to patellar sesamoid development and function in adult ostriches, finding that high shear stresses around the distal femur may help explain why two patellar sesamoids form there.

### Application of FEA to soft tissue biomechanics

FEA is also used for studying soft tissue biomechanics in the context of locomotor function. Insights that are gained from FEA of muscles can be highly compatible with those from experimentally based fluoromicrometry, in terms of discovering how soft tissues function in locomotion. Micromechanical finite element models can inform upon the 3D or quasi-3D biomechanics of muscles. An analysis of the M. rectus femoris and M. soleus in rabbits indicated that, unlike fibre-based muscles, fascicle-based ones have extremely different shear moduli and transversely anisotropic deformations (Sharafi and Blemker, 2010). Another study focused on three forearm muscles in mice, testing the model against histological data, and discovered that the endomysium around fibres resists active muscle forces, passively regulating strain in the terminal ends of fibres and thus potentially playing a protective role (Sharafi et al., 2011).

Realistic 3D models of muscle can give deeper insights into the dynamics of, and interactions between, muscles during various tasks or with different geometries (Fig. 4). In many musculoskeletal models, muscles are represented as simple 3D lines of action, especially in earlier musculoskeletal modelling (i.e. Nagano et al., 2005; Hutchinson et al., 2005), sometimes including some wrapping behaviour. Recent methods, predominantly focused on humans, have enabled more complex modelling of muscle lines of actions during locomotion. For example, muscles can be modelled as finite element meshes in musculoskeletal dynamic simulations (e.g. Elyasi et al., 2022, who investigated human patellar movements). Blemker and Delp (2005) developed a finite element model incorporating muscle fibres: ‘fibre geometry templates’ were transformed to a volumetric muscle mesh to create muscle-specific geometries. Changes in muscle shapes as a result of joint rotation were accurately predicted by the 3D muscle fibre models in comparison to empirical MRI data (see also Blemker et al., 2007). Fibre-based FEA muscles have been integrated with musculoskeletal dynamic simulation (Lloyd et al., 2012; Stavness et al., 2012). Kohout and Kukačka (2014) and Modenese and Kohout (2020) developed a similar approach using fibre templates to generate curved fusiform muscles in a model. The muscle fibre displacements during movement were calculated by linking the transformations of each fibre point to the two closest bones, with the influence of each bone's position diminishing with increasing distance.

### Biomechanical models and simulations of neuromuscular dynamics during legged locomotion

**Fig. 5.**
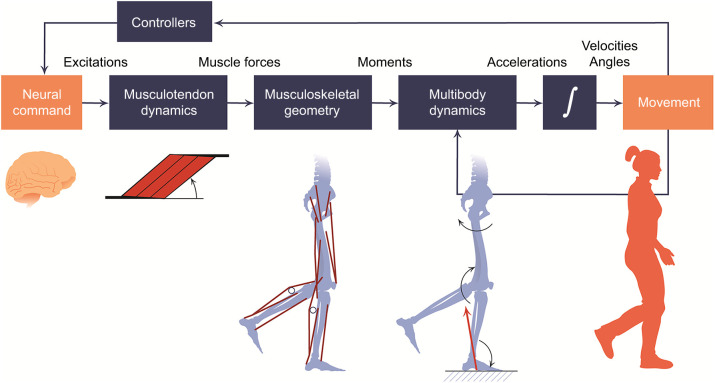
**Typical workflow in a 3D forward dynamic musculoskeletal simulation.** Workflow taken from Seth et al. (2018; https://doi.org/10.1371/journal.pcbi.1006223.g001 CC BY 4.0 license). Input data are controllers (optimisation criteria, constraints, etc.), assumed musculotendinous model (e.g. Hill; Millard et al., 2013) used for computing dynamics, and musculoskeletal geometry. Orange boxes emphasise the start and end points of the simulation; dark grey boxes are the intermediate steps. Running the simulation solves for the controls (neural commands) leading to muscle excitations and activations and thereby forces, generating joint moments that produce (via rigid body dynamics; multibody dynamics analysis or MDA) accelerations and other kinematics of locomotion. Outputs can then give insight into the underlying controllers, and across a stride(s) the changing movements will result in different initial conditions for computing the dynamics over the next time step.

Modelling and simulating terrestrial locomotion are powerful ways to test locomotor mechanisms. These methods involve a choice between simpler, inverse dynamic (typically static; see Glossary) approaches and more forward dynamic (even predictive; see Glossary) ones. These approaches are often somewhat misleadingly labelled ‘multibody dynamic analysis’ (MDA; see Glossary; Fig. 5) but MDA purely involves rigid body dynamics, not necessitating any biological data (e.g. vehicular crashes), unlike musculoskeletal models and simulations, the terms we use here (e.g. Seth et al., 2018). The inverse dynamic approach requires the following input data: external forces, body inertial properties, joints, muscles and kinematics – the latter may be motion data captured by XROMM as discussed above. Kinematics and kinetics may be assumed (e.g. where unknown, such as for an extinct taxon; e.g. Bishop et al., 2021c), or track specific experimental data. This approach produces a rigid body mechanical framework (i.e. MDA) to which muscle models are appended (e.g. Millard et al., 2013), and joint moments and muscle activations are extracted. Forward dynamic simulations have a different setup, in which external forces and kinematics are not used as input data (Fig. 5). Rather, only inertial properties, joints and muscles are used to create a musculoskeletal model. Control targets and cost functions may define an optimal control (see Glossary) problem to produce optimal muscle activations, simulated forces (via MDA) and simulated kinematics. For example, a predictive simulation (see Glossary) seeking to ascertain the maximal jumping height of an animal (e.g. Bishop et al., 2021d) would first dictate constraints on the task (e.g. bounds on joint angles), a time to accomplish the task (e.g. 1.5 s), and a target to be optimised (e.g. jumping as high as possible). Further constraints/cost functions can also be appended, such as minimisation of muscle activation.

### Predictive simulations in palaeontology

Studies have used optimal control methods in predictive simulations of locomotion in extinct taxa to answer questions about locomotor behaviour, which is important as experimental data are entirely absent for fossils. These methods are best evaluated by first simulating movement in an extant species (more so if *ex vivo* or *in vivo* data are available) and then extrapolating the approach to an extinct analogous species. For example, Bishop et al. (2021c) used a musculoskeletal model of a tinamou, formulating an optimal control problem of generating a gait cycle which was solved by constraining a target forward speed. This produced a minimal walking speed and a maximal running speed, which formed the baseline for simulating the maximum running speed in the extinct dinosaur *Coelophysis* – and further highlighted the important 3D dynamic contribution of the tail to regulating angular momentum during movement.

More theoretical biomechanical simulations can predict or otherwise test locomotor behaviours that are not directly observed. Studies by Sellers and colleagues have used evolutionary algorithms (solving an optimisation problem by applying concepts of biological evolution to ‘generations’ of potential solutions) to predict locomotor dynamics in a variety of species, such as suggesting that diagonal footfall patterns are not chosen based on simple optimisation criteria in quadrupedal chimpanzees but rather are likely to be a compromise between energetic efficiency and lateral stability (Sellers and Hirasaki, 2018). Sellers et al. (2022) generated plausible neuromechanical simulations (i.e. incorporating neural control) of non-steady locomotion such as stopping and turning. Simulations of dinosaur locomotion have given insights into their maximal locomotor performance and gait dynamics, such as slow walking and low joint mobility for a giant sauropod (Sellers et al., 2013); and a relatively slow ‘grounded running’ gait for *Tyrannosaurus* (Sellers et al., 2017). Surprisingly fast bipedal and quadrupedal gaits for the fairly large ornithopod dinosaur *Edmontosaurus* intimated that unrepresented constraints might be causing overestimation of locomotor performance (Sellers et al., 2009).

### Synergistic integration of empirical and theoretical methods

**Fig. 6.**
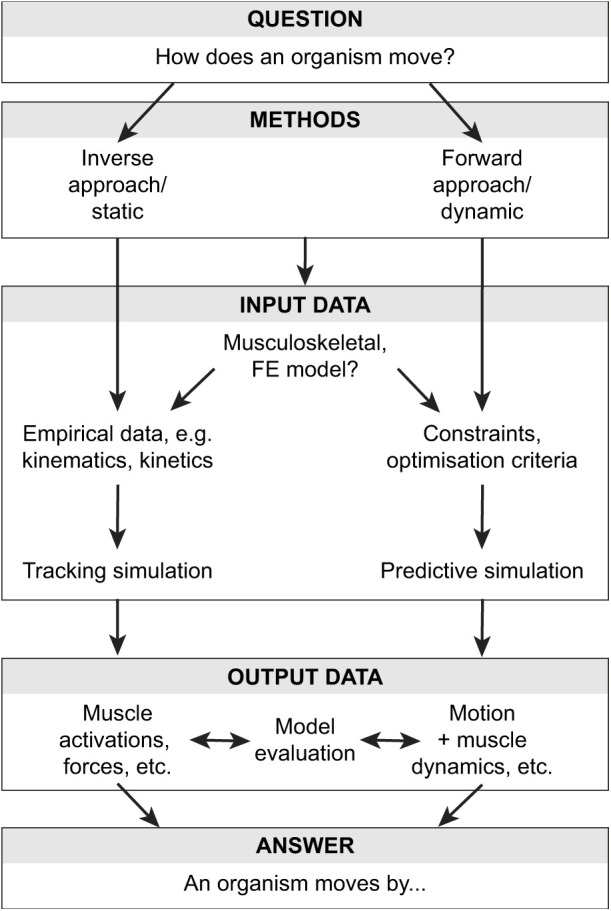
**Generalised workflow for study design and conclusion using various integrative 3D digital simulation methods.** Choices arise between more inverse or forward dynamic methods, then what kinds of tissue models are used [finite element (FE) and/or musculoskeletal], and how much empirical/theoretical data are integrated, leading to analyses via, for example, tracking/predictive simulation that produce data used in model evaluation, and then ultimately an answer to the study's question(s).

The combination of empirical and theoretical approaches (‘synergistic’; Fig. 3) can address research questions that are difficult to answer with either approach on its own (Fig. 6). This combination can be achieved using empirical data as per above, or using robotics or fluid dynamics approaches. A variety of studies have combined empirical data with simulations to study 3D locomotor dynamics – including prediction of jumping performance in frogs (Kargo et al., 2002; Porro et al., 2017; Richards et al., 2018; see also Collings et al., 2022), joint reaction forces and soft tissue strains as well as substrate dynamics in horse forelimbs (e.g. Swanstrom et al., 2005; Becker et al., 2019, 2020; Harrison et al., 2010; Symons et al., 2016, 2017), neuromuscular control in rats and mice (e.g. Johnson et al., 2011; Yeo et al., 2011; Charles et al., 2018), muscle activations in dog forelimbs (Stark et al., 2021), and relative postural leverage or ‘effective mechanical advantage’ across the evolution of giraffids (Basu and Hutchinson, 2022). Neuromechanical analyses of walking in cats have benefitted from integration of empirical- and simulation-based data for the hindlimbs. Simulations have shown how feline hindlimbs produce forces in postural control mechanisms that resist perturbations (McKay et al., 2007), how muscles are activated to couple joint dynamics proximodistally in limbs (van Antwerp et al., 2007) and how the muscular anatomy influences constraints on the coordination of limb endpoint forces (Bunderson et al., 2010), demonstrating that inverse simulations produce reasonable matches to experimental *in vivo* data from electromyography (EMG), MTU lengths and tendon force–buckle measurements (Karabulut et al., 2020). Quite a few of the above examples are more inverse dynamic approaches than forward dynamic, whereas truly predictive (without much empirical data, but still realistic/synergistic rather than conceptual) simulations remain rarer.

**Fig. 7.**
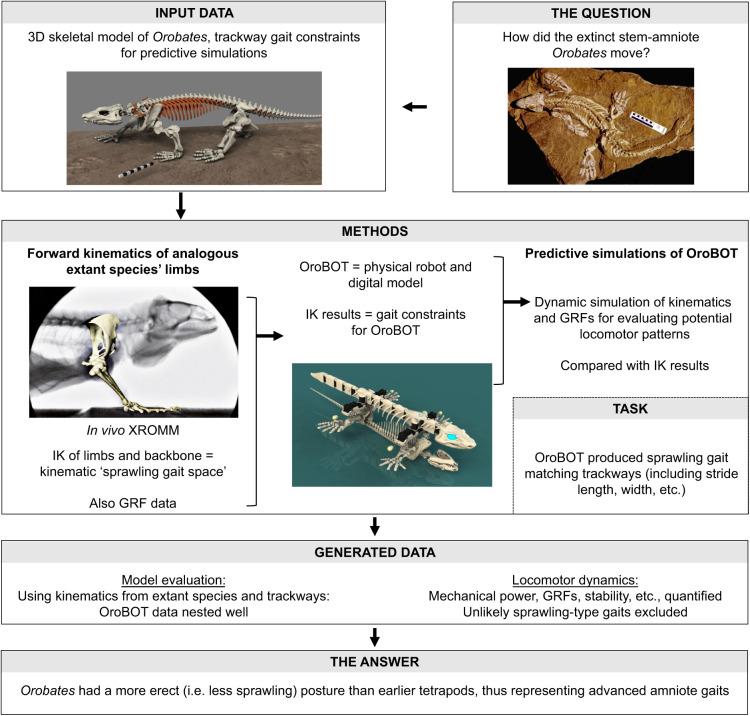
**Example integrative 3D digital research study workflow.** Workflow adapted from Nyakatura et al. (2019). Images used with permission, courtesy of (clockwise from top left): Thomas Martens; Jonas Lauströer, Amir Andikfar and John Nyakatura; Konstantinos Karakasiliotis; John Nyakatura.

Digital empirical and theoretical approaches can also be integrated with, or tested against, digital and physical robotics approaches (Karakasiliotis et al., 2016), even including palaeontological and evolutionary inferences. Building on prior 3D bio-inspired robotics studies by Ijspeert et al. (2007), Nyakatura et al. (2019) investigated how the early amniote tetrapod *Orobates* walked by combining (1) experimental XROMM and ground reaction force (GRF) data from four extant tetrapods used to constrain a kinematic simulation of *Orobates*, (2) both dynamic simulation and robotic representations of *Orobates*, and (3) a ‘sprawling gait space’ of 3D kinematic solutions that would fit the actual fossil trackways of *Orobates*. Together, these analyses pointed toward a derived, somewhat erect (more crocodile-like than salamander-like) limb posture for *Orobates* (Fig. 7). There are relatively few studies that have conducted such 3D integration of rigorous morphology-based experimental kinematics (and kinetics) with simulations and robotics. As Nyakatura et al. (2019) showed, there is great similarity between physical (hardware) robotics approaches and digital models and simulations (‘virtual robotics’) – including those used by roboticists – and these two methods have great synergy in biomechanics despite their differences (e.g. theoretical simulations produce data not obtainable by physical robot experiments; the latter generate actual locomotion in the real world). Together, those data can test hypotheses about what behaviours or performance could be generated by certain morphologies, or how those are controlled.

Integration of biomechanical experiments and models and simulations with digital fluid dynamics analysis has seldom been conducted in studies of tetrapod terrestrial legged locomotion, but is powerful for addressing the mechanistic influences of rigid body and fluid dynamics. Kinematics of the wings from XROMM, force platforms (hindlimb GRFs) and aerodynamic forces from *ex vivo* propeller experiments were tracked by Heers et al. (2018) in inverse simulations. These simulations predicted wing muscle forces and activities, with reasonable matches to empirical muscle excitations, for three ontogenetic stages of chukar birds while they conducted wing-assisted incline running. However, no studies to date have fully integrated 3D digital fluid dynamics (e.g. computational fluid dynamics) into such analyses. Regardless, there is exciting potential shown by studies such as that by Falkingham and Gatesy (2014), where 3D discrete element method simulations of substrate deformation in ‘sediments’ represented by poppy seeds (and rigid substrates) were compared (and integrated) with XROMM data for walking guinea fowl birds, and their real tracks. These particle-based analyses (building upon the groundbreaking 3D kinematic study of Gatesy et al., 1999; also see Falkingham et al., 2020) not only demonstrated how 3D footprint shapes were produced during a complex mechanism that is otherwise invisible but also showed homologous traits of track formation via comparisons with fossilised theropod dinosaur tracks.

### Combination of musculoskeletal simulations and FEA

Integrating musculoskeletal models with FEA enables investigation of how bones mechanically respond to locomotor loads primarily imposed by muscles, and how bony or muscular morphology affects this mechanical response (Fig. 4). Historically, musculoskeletal simulation and FEA have been combined by running simulations, then using resulting muscle forces or joint reaction forces as inputs for FEA simulations in separate programs, but more recent developments conduct the two analyses in the same model (e.g. Lloyd et al., 2012). As an unusual example of integration of musculoskeletal simulation and FE models, Goetz et al. (2008a,b) built a 3D model of an emu hindlimb and used the simulation results in a thermal finite element model of the femoral head to simulate loading-induced bone necrosis. Shahar et al. (2003) applied loads informed by experimental GRF and kinematic data and a musculoskeletal model to a 3D finite element model of a canine femur to estimate stresses and strains, showing that joint reaction forces alone overestimated these values and that, if muscle forces were included, peak values in the diaphysis were medial (compression) and lateral (tension), indicating bending. A variety of studies have applied FEA to horse limbs, especially feet, along with 3D empirical data (e.g. XROMM) and musculoskeletal simulations (e.g. Harrison et al., 2014; Panagiotopoulou et al., 2016), generally aiming at more veterinary clinical applications. Bishop et al. (2018) devised a different kind of integration, whereby a musculoskeletal simulation of a chicken hindlimb was used to calculate muscle forces for application to a finite element model, testing how the principal stress trajectories compared with actual cancellous bone architecture. They discovered a good correspondence, supporting the usage of cancellous bone structure to infer general hindlimb orientation in theropod dinosaur hindlimb bones, and the evolution of limb poses. A 3D musculoskeletal model of a rat hindlimb using XROMM kinematic data in an inverse simulation of locomotion produced data that compared favourably with *in vivo* experimental EMG data and bone strains in the femur (Wehner et al., 2010).

## Pitfalls and challenges

Uncertainty in models and simulations often complicates the testing of hypotheses about locomotion. These uncertainties arise from many aspects of design and implementation: imprecision or inaccuracy in empirical inputs; oversimplification hiding important effects; errors in computational implementation; or the inappropriate mathematical or computational implementation to the problem of interest. In making inferences, there must always be a ‘leap of faith’. If we could directly measure the phenomenon of interest, or knew the ‘answer’ to the question already, we would not need a model. However, we can increase trust and credibility in a modelling framework through verification (see Glossary) and ‘validation’ (a misleading term as this implies rendering a model infallible or 100% accurate, when all models are ‘wrong’). As covered in detail by Hicks et al. (2015), model evaluation (Fig. 6; see Glossary) begins from the formulation of the research question – asking whether the model can answer the research question of interest, and whether the modeller understands the assumptions being made. It continues with comparing the model and simulation with well-established results, either from empirical data or from other models. Robustness and precision (see Glossary) can be evaluated through sensitivity analyses – examining how input parameter changes affect outputs (Hutchinson, 2012). Finally, the careful documentation and dissemination of methods, data and software used allows peers to test, evaluate and expand models beyond the life of a single publication.

Model evaluation can be difficult with high-dimensional models and data, which are more common in 3D digital methods. The problem is exacerbated when increasing biological fidelity is sought. As an example, if the research question was about a limb's 3D dynamic mechanisms in locomotion, then we would first need to model an adequate amount of DOF and muscles. However, for every dimension that is added, there is in turn an increasing number of possible solutions. Challenges from additional joint DOF (many dozen in one real limb; e.g. Manafzadeh et al., 2021) are compounded by adding muscles, which would number several dozen in one limb. Excessive dimensions inevitably will significantly increase simulation time and could make a simulation computationally infeasible as a result of the exponential growth of variables – the so-called ‘curse of dimensionality’ (e.g. Halilaj et al., 2018). Rather, it is necessary to reduce the number of input variables to generate results, whilst maintaining sufficient realism to answer the research question, i.e. finding a suitable balance. The reduction of dimensions introduces a level of abstraction to models and simulations whose consequences can be difficult to evaluate (Hicks et al., 2015). Simulation data can of course be compared with existing data, such as simulated muscle excitations in comparison to EMG data (Bishop et al., 2021a,b,d), but existing data can be limited and challenging to obtain. Sensitivity analysis of the effects of model complexity on simulations are extremely valuable. For example, Kargo et al. (2002) conducted a sensitivity analysis of a frog hindlimb to find the minimal DOF required to adequately simulate jumping performance.

Another challenge is data overfitting (see Glossary), which can give the researcher the false security of inflated model accuracy (see Ferber et al., 2016). Two possible scenarios can be envisaged. First, results may become too similar, reducing a realistic range of data present within the variable, and errors may become harder to spot. Second, if numerous models and/or conditions are modelled via overfitting, the researcher may inadvertently fail to acknowledge errors which would otherwise be easily spotted without overfitted data. Human biomechanists have attempted to solve such issues via developing trained neural networks (Smirnov et al., 2021) and machine learning tools (Halilaj et al., 2018; Song et al., 2021), but as yet such developments have not been implemented for models of many other animals. These issues will be present in simulations using trained data as feedback loops, but evaluation of the results should minimise the risk of overfitted data in such circumstances (Hicks et al., 2015).

## 3D analysis: caution and potential

Returning to an issue raised in the Introduction, future studies should continue to ask themselves during study design and perhaps explicitly within their publication: why is a 3D approach required, and why is the level of complexity therein required, to answer the research question (Figs 3 and 6)? As computational power inevitably increases (e.g. Mack, 2011) and 3D tools become more sophisticated, more complex 3D questions can be answered – but is that necessary? Weighing necessity versus sufficiency remains important, and is not so often explicitly addressed in 2D or 3D studies. Real organisms have complex 3D morphology and move in (very) complex 3D environments, but most studies may not need to maximise complexity. Furthermore, some added complexity might not even be biologically accurate, or remains debatable. An example is the practice of breaking single line-of-action muscles into numerous strands (described above). Whilst this is geometrically more realistic in 3D, and might provide better finite element results, a question remains how to activate these strands in a biologically realistic way – should the activation of separate fibres be homogeneous or heterogeneous? This remains a frontier in which answering its fundamental questions will require more integration with neuroscience. More generally, we worry that the attitude of ‘more complex and realistic is always better’ is very dangerous (see also ‘Pitfalls and challenges’ above, especially the ‘curse of dimensionality’). Researchers might be seduced by the attractiveness of 3D approaches, but gradually these approaches may provide more and more incremental answers as they are applied to the same or similar question(s). This danger could be especially risky for early career researchers, who should be appropriately mentored in choosing and wielding these complex tools.

On the more positive side, there is a vast vista of unanswered, exciting questions in this field that could benefit from the application of these 3D approaches, and innovations of them, as the techniques rapidly mature. New discoveries, beyond science's capacity less than 20 years ago, could thereby be made regarding how locomotion functions in particular species and evolutionary lineages, as well as the fundamental mechanisms, constraints and principles underlying this locomotion. Empirical approaches will always have great value in measuring important parameters, but we can never measure everything. Theoretical approaches have their well-known limits, but can estimate that unmeasurable information – or they can predict things we might not have expected. Together, these approaches have much synergy for testing major hypotheses (Figs 3, 6 and 7). Furthermore, these approaches not only can answer how individual organisms or species move but also can influence other fields, particularly fields close to comparative biomechanics such as physiology, engineering/robotics and evolution/palaeobiology, but also fields that overlap with these, such as ecology (e.g. the role that 3D locomotion plays in ecosystems) and environmental sciences (e.g. the role that 3D environments play in locomotion).
